# Hospital and Institutional News

**Published:** 1917-12-22

**Authors:** 


### HOSPITAL AND INSTITUTIONAL NEWS.

### KING EDWARD'S HOSPITAL FUND.

THE evolution, progress, and triumph of King Edward's Hospital Fund is one of the most wonderful, as it is an entirely gratifying example of what the metropolis of the Empire can accomplish when it wills to do well. On page 249 of the present issue onwards will be found a full account of the annual meeting of the Governors and General Council of this Fund at St. James's Palace. Everyone interested in hospitals—and who is not?—should read it and keep it. For the Fund has given away this Christmas no less than £190,000, which constitutes a record. His Majesty's letter is one more testimony to the undeviating and generous interest taken by King George in the triumph of the voluntary system of hospitals in this country. The Speaker's address makes some telling points of momentous importance, and its allusion to King George's Fund for Sailors is welcome for two reasons—first, that Fund is modelled and worked on the plan of King Edward's Hospital Fund; and secondly an attempt has been made to throttle its energies and damage the Christmas budgets of the majority of seamen's charities by one enterprise, the action of which is as meretricious as it is wholly without justification. It is gratifying to note that King George's Fund, after only five months, has proved to be a record success—a fact which will be welcomed by the Nation and Empire.

### THIS WEEK'S ILLUSTRATIONS.

WE publish again this week more illustrations from the Exhibition at the Camera Club of original black-and-white drawings by patients and staff of the 3rd London General Hospital, Wandsworth. The exhibition will remain open till December 31 at 17 John Street, Adelphi, Strand, W.C., near the Little Theatre, and everyone who can spare a few minutes should not fail to pay it a visit. Our obligation to the Exhibition Committee, the Editor of the *Gazette* of the 3rd London General, and to the ever-courteous and indefatigable Lance-Corporal Ward Muir is great. We are much in their debt and full of gratitude for their generous co-operation.

### DR. GARRETT ANDERSON.

BY the death of Mrs. Garrett Anderson there has been removed the foremost pioneer of the practice of medicine by women. Although she was not the first woman to be placed on the British Medical Register, for Dr. Elizabeth Blackwell, who had obtained a medical degree in the United States, held this honour, yet Dr. Garrett Anderson was the first woman to obtain in this country a medical diploma. In 1865, when she was Miss Garrett, she passed the examination of the Society of Apothecaries of London, and this qualified her to practise medicine in the British Isles; five years later she obtained by examination the degree of M.D. of Paris. Yet in a way her success stimulated those who opposed the admission of women to the medical profession, and several years had to pass before most of the hindrances were removed. She was one of those who collaborated with the late Dr. Jex-Blake to found the London School of Medicine for Women, the pioneer institution in this country for teaching the medical sciences to women: to her energy also was mainly due the founding of the New Hospital for Women, though it is hardly "new" now, for it has recently celebrated its fiftieth anniversary. For many years she was Lecturer on Medicine at the School of Medicine for Women, for long she was its Dean, and at the time of her death she was its President. She did much to enable women to study medicine, and it is largely due to her energy that now it is as easy for a woman as for a man to become a medical practitioner. Her daughter, Dr. Louisa Garrett Anderson, organised the first military hospital staffed by women, and she received one of the first appointments to the new Order of the British Empire.

### THE CASE OF BAGULEY SANATORIUM.

IT is difficult not to sympathise with the difficulties of the medical superintendent of Baguley Sanatorium, which are tersely set out in his latest report. With the opening of the new wards the sanatorium, when full, accommodates 188 men and 130 women. The new wards are so "open" that the medical superintendent describes them as "most unsuitable for the type of case we get at Baguley," which is the more serious because the type includes "men from the Army who have broken down on active service, some of whom have undoubtedly had the resisting power of their lungs weakened by an attack of gas poisoning." In consequence, the death-rate was 18.2. Then comes the crux of his complaint: "Such a large establishment, filled with patients suffering from a chronic disease and each having his own particular crotchet, with only one assistant to help, absolutely prohibits anything being done in the way of research or special treatment for individual cases. These two things, research and special forms of treatment, are the only inducements which can be offered to medical men to come to a place like Baguley, which is to a large extent simply an isolation hospital." There is a directness and a conviction in the presentation of this report which make it a model in its way. The facts are stated, and they stick like burrs.

### THE SIR EDWARD WOOD MEMORIAL.

To complete in fact, though not according to the original plan, the big reconstruction of Leicester Royal Infirmary as a memorial to the late chairman, Sir Edward Wood, is the design of those who wish to perpetuate his memory. This will mean to rebuild the wing which contains the St. Luke and accident wards. The cost is expected to be £30,000. The Committee in charge has invited contributions in the form of Five Year War Bonds, and the Hospital Saturday Society of Leicester is seeking to enlist its members in support of the memorial, which, after all, is in honour of the man who was the first president of the society. The architects, Messrs. Everard, Son and Pick, propose that the south-west wing when reconstructed shall contain a total of ninety beds, thirty more than it has at present, together with a nurses' dining-room, a sewing-room and store-rooms. Upon the upper floors, where the wards will be placed, each ward unit will include a clinical laboratory. The main scheme, when carried out, will complete for the present generation the long task on which Sir Edward Wood set out, and three-quarters of which he lived to see accomplished under his own chairmanship.

### MR. H. H. WILLS.

The new president of the Bristol Royal Infirmary, Mr. Harry H. Wills, who, after Sir George White's death, was too much occupied to accept the office till last week, has reconsidered his decision. He has asked to be relieved, however, of acting as chairman of the committee and of the treasurership, which the honorary secretary is therefore undertaking. The new president begins with a less heavy financial prospect than that which confronted his predecessor, and his election is another link between his family and the public life of the city.

### THE MANCHESTER RADIUM COMMITTEE.

It is good to hear that the institute over which this committee presides is now paying its way, and that it possesses the £1,000 required to complete the laboratory's equipment. Sir Edward Holt is now to apply for official recognition, so that the institute may be placed on a par with other hospitals engaged upon military work. Soldiers and discharged men form a large proportion of the patients, but the difficulty of maintaining the staff is so great that the committee has been unable to allow Dr. Arthur Burrows, its pathologist, and Mr. Hartley Lupton to apply for commissions in the Royal Army Medical Corps. In addition to an investment of £5,000 in War Stock, the Committee has nearly £700 in hand, and expects to close the year with a balance of £300.

### WHAT IS THE VIRTUE OF AN HONORARY SECRETARY?

Except in the case of small hospitals we have never seen the advantage of employing an honorary secretary. The practice, however, still survives, even though a paid assistant is usually necessary. We were not surprised to hear recently that an institution with sixty-five beds, which wishes to maintain the practice, was unable to secure the services of any man to undertake the post. A woman was therefore appointed temporarily. What are the advantages of an honorary secretary in an institution of any size?

### THE TRUTH ABOUT GERMAN HOSPITALS.

It is not always the mayor of a town who is against the occasional proposal for the State support of voluntary hospitals. Mr. T. G. Small, the Mayor of Nottingham, lately expressed himself very strongly in support of the voluntary system. His criticism was directed against the proposed National Insurance Organisation. He was followed by Mr. Louis F. Pearson, who remarked that "qualified Englishmen" had visited State-owned hospitals in Germany before the war, and had found the buildings, indeed, finer, but the attention to patients and the atmosphere of the institutions much below the standard even of our workhouses. We may recall that The Hospital published before the war a series of articles upon the German hospitals by Sir Henry Burdett, in which an impartial survey was given of the fine buildings and admirable gardens on the one hand, and the inhuman system of scientific callousness which prevailed in them on the other. At the time the articles were written there was no anti-German bias. In fact, any bias that existed was rather the other way. One "qualified Englishman" recorded his impressions in time.

### MOTHERS' PENSIONS AND JUVENILE CRIME.

We publish this week a letter from the State Children's Association suggesting an alternative to the imprisonment which is now being meted out to youthful offenders. The proposal suggested can be studied on another page. But we should not have admitted the letter had the State Children's Association not a much better argument, the value of which, though coming from another source, has already been emphasised in The Hospital. On the postulate that the real cause of what is rhetorically called "the tide of lawlessness" among boys is simply the absence of their parents on military service or war work, the omitted argument affirms that the establishment of mothers' pensions, on the lines which an American judge has lately made familiar, by keeping the mother at home would do something to supply the father's absence. Now we are assured by the State Children's Association that it is trying to establish a mothers' pension scheme, and, further, that the Association believes that "such a scheme will greatly decrease juvenile delinquency." It is worth trying, at all events, and such a scheme promises better than any forced friendship among people of different tastes and pursuits, which, in the absence of a genius for this sort of thing, rarely does more than educate, possibly by a rebuff, the elder person. To try to keep the home together is the proper plan.

### WHAT CAN BE DONE IN A YEAR!

We propose to vary our recent references to the remarkable set of appeals which Mr. Leonard Rea has been addressing to the people of Cardiff by a terse summary of the new developments which have actually occurred during the past year at King Edward VII.'s Hospital. A new and elaborate orthopædic department has been opened; a preliminary training-school for probationers, "Anthony House," has been opened; the Lee Convalescent Home for Women and Children at Lavernock has also been opened. In addition, the hospital's school of massage and Swedish exercise and its electrical department have been established. The conditions of service for nurses and their syllabus of training have both been revised. This is an astonishing record. But General Lee and Colonel Bruce-Vaughan do not rest from their labours, for they set themselves an enormous task, and determined to complete it in the shortest possible time. To convert Cardiff Infirmary into King Edward VII.'s Hospital was to develop a provincial institution into one of metropolitan rank—the centre, in fact, of a great medical school. The Elize Nixon Flat, with its twenty maternity beds, will be ready for work in January, together with the temporary building in Glossop Road, designed for a similar number. The latter is a prelude to Sir Edward Nicholl's foundation. To maintain the flat an income of £6,000 a year is wanted, and the women of Wales are asked to give it. Why not, when they remember that Colonel Bruce-Vaughan still admits a waiting-list of 1,000 cases, among whom are now many expectant mothers? The measure of work *undone* explains the need which makes Colonel Bruce-Vaughan indefatigable.

### PROMOTION IN THE I.M.S.

A quite legitimate grievance of officers in the Indian Medical Service will, it seems, be speedily rectified. There has been, for some time past, considerable discontent arising from the fact that, whilst in the R.A.M.C. temporary officers rise to captaincy automatically after one year's service, three years, or the old pre-war rate of promotion, elapse before a lieutenant in the I.M.S. attains his promotion. By a recent Order in Council the Secretary of State for India has adopted the following special measures regarding the promotion of officers in the Indian Medical Service: Lieutenants promoted up to December 31, 1916, will be promoted to captain, with effect from the completion of one year's service. Previous military service rendered after July 16, 1915, will also count towards promotion, though no promotions will be ante-dated further back than September 1, 1915. Pay and allowances will be allowed from September 1, 1916, but promotions under the new scheme between this date and September 1, 1915, will be effective for purposes of wound, injury, and family pensions or gratuities. Captains are also to be ante-dated, with the view of securing for them a relative equality in seniority with that of corresponding members of the R.A.M.C., whose promotion has been specially accelerated by war conditions. On the conclusion of the war officers will not be eligible for further promotion until they have completed the period of service that would have been necessary had their progress not been accelerated by war conditions.

### CLERK TO THE GOVERNORS OF GUY'S HOSPITAL.

Mr. J. Curry, clerk to the governors of Guy's Hospital, appeared at Southwark Tribunal, at the request of the National Service representative, to show cause why his certificate of conditional exemption should not be withdrawn. His appeal for further exemption was strongly supported by Sir Cooper Perry, the superintendent, who wrote pointing out that Mr. Curry not only attended the quarterly meetings of the governors, but also of the various committees. He also dealt with the financial department of the hospital, which included the supervision of £45,000 received in endowments and £45,000 in voluntary subscriptions every year. Mr. Curry was responsible for organising appeals for funds; was manager of *Guy's Hospital Gazette*; and secretary to the Clinical Research Association, Limited. Mr. T. Haynes, J.P., said he knew the valuable work Mr. Curry was doing for the hospital. The conditional exemption certificate was withdrawn, but the National Service representative said he should not object to applicant being granted six months' exemption instead, in view of the splendid work he was doing for the hospital. The Tribunal decided to grant Mr. Curry six months' exemption.

### RED CROSS WORK AFTER THE WAR.

It was whispered before the meeting of the Hospital Officers' Association at the Royal Society of Arts on the 14th instant, that Sir Arthur Stanley, in his address on Red Cross work, might say something new and interesting about the relationship of the Red Cross Society and voluntary hospitals after the war, and his hearers were not altogether disappointed. What was said was not very definite, and necessarily was qualified by the date of peace, and, consequently, the extent of Red Cross funds on this happy day, but Sir Arthur did clearly express the hope that some means would be evolved of continuing the work of the British Red Cross Society and the Order of St. John of Jerusalem, in conjunction with voluntary effort, for the general welfare of the sick and suffering. How greatly they would be able to help was difficult to say. Funds were coming in well, "Our Day" was already in excess of the million achieved in 1916, but, as the rate of expenditure, owing to new and large calls, was at present £5 per minute, and would very likely reach £6 or £7, it was clear that the public must be asked to continue to give largely. Sir Arthur dealt with the obligations that the Societies had taken in respect of certain groups of discharged and disabled men, such as the paralysed, neurasthenics, epileptics, and cases of advanced tuberculosis, and said that it was hoped that their aid towards solving the problem of dealing with these patients would be continued after the war.

### LLANELLY AND INFANT WELFARE.

Few small towns have done more in the way of infant-welfare work than has Llanelly, and this is largely due to the energy and enthusiasm of Lady Howard, who is an alderman of the borough council. It is due to Lady Howard's efforts, for instance, that the obnoxious long-tubed feeding-bottles for babies are being banished from Llanelly, for it was she who succeeded in obtaining the co-operation of the local chemists in the discouragement of would-be purchasers of them. At a meeting of the Llanelly Council last week Lady Howard was pleading the necessity for increased accommodation for infant-welfare work when another alderman—a male member, of course—threw cold water on the proposal by referring to "fads and faddists," and declaring against the removal of expectant mothers to a maternity home. Lady Howard was not dismayed by such opposition, and carried her point, the matter being referred to the estates committee.

### A GRANT FOR PLAY CENTRES.

At a recent meeting of the Sunderland Town Council a proposal was brought forward by the Education Committee that a grant of £1,800 per annum should be made to the Child Welfare Association for the establishment of twelve play centres for children in the borough. An amendment was moved that the minute be struck out, it being contended that if the expenditure had to be incurred the money should be spent by the Education Committee, and not by "an irresponsible body." The Council, however, did not accept this view, deciding to make the grant as suggested.

### A MONSTER THISTLE.

The second issue of the souvenir book of the Scottish Women's Hospitals engaged in foreign service at Salonika and in France, Serbia, Corsica, and Rumania is a monster magazine. The *Thistle*, as it is called, is more ambitious in size and various in contents than anything of its class which we have seen. For eighteenpence its readers are provided with 100 handsomely printed pages, apart from dozens of illustrations both in colour and black-and-white. President Poincaré contributes an autographic benediction, and the editors of the literary and pictorial sections have taken evident trouble to find original work and to satisfy diverse tastes. Tucked away, for instance, among tales and articles, verses, and even songs set to music, is a reverie by Mr. Forrest Reid, who deserves mention because he is one of the few younger writers who take an artistic, a professional view of literature. He is always trying to write well, to follow Pater's advice in using English as if it were a learned language, and must be respected accordingly. Mr. W. W. Gibson and Mr. de Vere Stacpoole contribute verses. In a charming metre Sir Owen Seaman addresses lines to "Belgium in Exile," whose only fault is an odious reminder of "The Rosary." The illustrations would require an article to themselves. The whole is an excellent number, with the lavish display we have learned to expect from the N.U.W.S.S., its promoters.

### THE "BRITISH PRISONER OF WAR."

The latest war newspaper is the *British Prisoner of War*, which the Central Prisoners of War Committee will publish on January 1, 1918, and each succeeding month. The new journal will contain all available information concerning prison regulations, articles on prison camps, with maps and illustrations, reviews of books about prisoners, and even such camp notes as may be obtainable. Care Committees are invited to induce their subscribers to take the paper. Its price will be 3d. a number, or 3s. a year. The office, we understand, is at 4 Thurloe Place, S.W. 7.

### NO BEDS FOR DIPHTHERIA PATIENTS.

Dr. Lionel Baly, the medical superintendent of Lambeth Infirmary, has drawn the attention of the Board of Guardians to two cases where he was obliged to admit children suffering from diphtheria to the infirmary. The private practitioner attending them, having failed to obtain their admission to an isolation hospital belonging to the Metropolitan Asylums Board, reported the cases to the relieving officer. They were informed that there were no vacancies for cases of diphtheria, and under no circumstances could they be dealt with that day, but might be removed the following day. As there were fourteen other children in the house, and as it was urgently necessary for the two children already infected to receive special treatment, they were admitted to the infirmary and isolated as far as possible in a small ward. Dr. Baly pointed out that the admission of such cases to the infirmary caused great inconvenience, inasmuch as the ward and the ambulance had to be disinfected, and a special nurse supplied to deal with these cases. The question of providing additional isolation wards, which was already before the board, for ordinary circumstances, would become a matter of extreme urgency. The board, in deciding to draw the attention of the Metropolitan Asylums Board to these cases, stated that they were of opinion that the Guardians should not be called upon to provide accommodation in their institutions because of the failure of another authority to deal with the cases for which it was responsible.

### THIS WEEK'S DRUG MARKET.

Business is naturally very quiet, and dealers are beginning to think of stocktaking; in view, however, of the shortage of supplies, this is not likely to be such a lengthy process this year. The recent considerable buying of saccharin has naturally had the effect of reducing supplies, with the consequence that the price tendency is now in an upward direction. A large business has been done in honey at figures substantially in advance of the enormous figures previously paid. Also glucose is again dearer. Very high prices have been paid for orris root. Liquorice juice is snapped up at extraordinarily high prices. Other drugs which are dearer include ammonium carbonate, colocynth, sugar of milk, pepsin, antifebrin, formaldehyde, valerian, and artificial oil of wintergreen. On the other hand Cape aloes is rather lower in price, as also are resorcin, menthol, and phenacetin. At the moment there is no change in the quotations of salicylic acid and sodium salicylate, but in view of the fact that importations of these drugs from the United States are likely to become less and less it is not improbable that we shall see higher prices; fortunately English makers are increasing their output substantially, and this factor will act as a brake on the market. The price of aspirin is not so well maintained.

